# Lemmel Syndrome: A Case Report

**DOI:** 10.7759/cureus.110342

**Published:** 2026-06-06

**Authors:** Micaela N Sousa, Teresa G Rocha, Pedro Simões

**Affiliations:** 1 Department of Internal Medicine, Unidade Local de Saúde do Nordeste, Bragança, PRT; 2 Department of Internal Medicine, Unidade Local de Saúde de Vila Nova de Gaia/Espinho, Vila Nova de Gaia, PRT

**Keywords:** ampulla vater, common bile duct, lemmel syndrome, obstructive jaundice, periampullary duodenal diverticulum

## Abstract

Lemmel syndrome is a rare and misdiagnosed cause of obstructive jaundice due to a periampullary duodenal diverticulum (PAD) in the absence of choledocholithiasis or a tumor. PADs are extraluminal outpouchings of duodenal mucosa that develop within a radius of 2-3 cm from the ampulla of Vater. They are largely asymptomatic, but rarely they can cause acute abdominal pain and obstructive jaundice due to the extrinsic compression of the common bile duct or pancreatic duct.

PADs are mostly asymptomatic, but complications can occur, such as hemorrhage, perforation, diverticulitis, pancreatitis, choledocholithiasis, cholangitis, jaundice, or intestinal obstruction. Treatment workup depends on the clinical presentation.

The authors present the clinical case of an 84-year-old woman who presented to the emergency department with jaundice, refusal to feed, vomiting, and prostration with a two-day duration. On physical examination, jaundice and tenderness in the upper quadrants were observed. Blood tests revealed anemia, leukocytosis with neutrophilia, and elevated aspartate aminotransferase, alanine aminotransferase, lactate dehydrogenase, and gamma-glutamyl transferase. Abdominal computed tomography showed ectasia of the intra-hepatic bile ducts and main bile duct, compressed extrinsically by a PAD. Due to the patient’s previous functional status, conservative management was decided.

## Introduction

Described for the first time in 1934, Lemmel syndrome is a rare cause of obstructive jaundice, with few cases reported in the literature. It is caused by a periampullary duodenal diverticulum (PAD) in the absence of choledocholithiasis or neoplasm [1]. Duodenal diverticula are saccular structures that originate from a combination of increased intraluminal pressure and intrinsic fragility of the muscular layer, which results in the herniation of larger vessels that suppress the intestinal wall [1]. When located within 2-3 cm of the ampulla of Vater, they are named juxtapapillary or periampullary, and these diverticula are associated with a higher risk of choledocholithiasis, due to the stasis prompted by the external compression [1-3].

The incidence of PADs increases with advancing age, and it may be observed in 10-20% of patients who undergo endoscopic retrograde cholangiopancreatography (ERCP). In the majority, duodenal diverticula are asymptomatic and found incidentally [1,4]. However, less than 10% are symptomatic [1] and can cause acute abdominal pain due to extrinsic compression of the main biliary duct or of the pancreatic duct.

## Case presentation

An 84-year-old woman with a past medical history of arterial hypertension, dyslipidemia, type 2 diabetes mellitus, osteoarticular disease, and dementia presented to the emergency department with jaundice, refusal to feed, vomiting, and prostration with a two-day onset. On physical examination, jaundice and tenderness in the upper abdominal quadrants were observed, with no other findings. Blood analysis revealed hypochromic anemia, neutropenia, and elevated alkaline phosphatase, aspartate aminotransferase, lactic dehydrogenase, gamma-glutamyl transferase (gamma-GT), and C-reactive protein, as well as elevated total and direct bilirubin. Pancreatic enzymes were within the normal range, as shown in Table 1.

**Table 1:** Laboratory test results on admission. MCHC: mean corpuscular hemoglobin concentration; RDW: red cell distribution width; AST: aspartate aminotransferase; Gamma-GT: gamma-glutamyl transferase; LDH: lactate dehydrogenase

Parameter (unit)	Result	Normal Range
Hemoglobin (g/dL)	9.8	12.3 - 15.3
Erythrocytes (x10^12/L)	3.3	4.1 - 5.1
Hematocrit (%)	30.1	35 - 47
MCHC (g/dL)	32.6	33 - 36
RDW (%)	18.5	11.5 - 15.0
Neutrophils (%)	41.5	50 - 70
AST (U/L)	58	< 31
Alkaline phosphatase (U/L)	618	30 - 120
Gamma-GT (U/L)	504	< 38
LDH (U/L)	528	< 248
C-reactive protein (mg/dL)	2.03	< 0.1
Total bilirubin (mg/dL)	2.43	0.3 - 1.2
Direct bilirubin (mg/dL)	1.10	<0.02

An abdominal computed tomography (CT) scan) with intravenous contrast was also performed, which showed ectasia of the intra-hepatic bile ducts and main bile duct, compressed extrinsically by a PAD, as presented in Figure 1 (coronal section) and Figure 2 (axial section).

**Figure 1:**
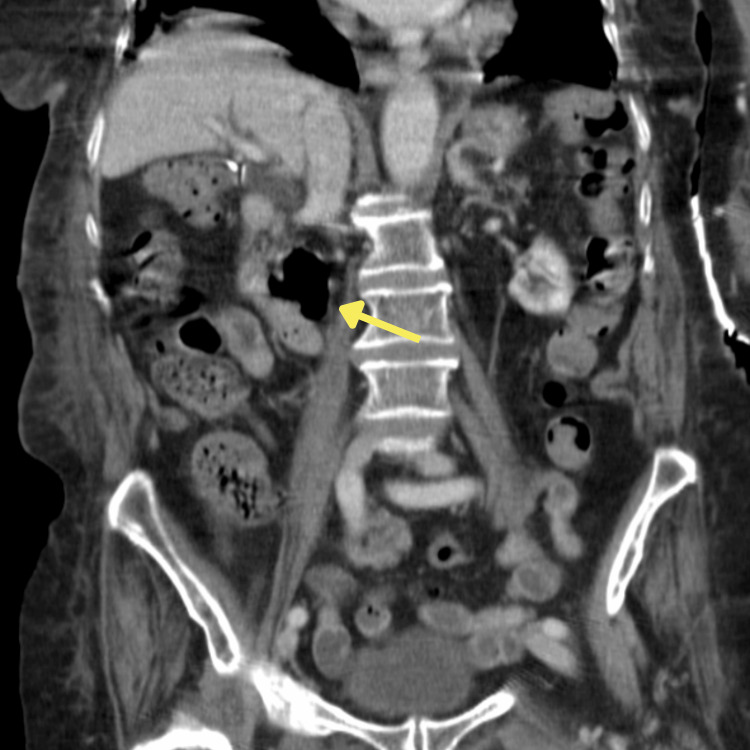
Coronal section of an abdominal computed tomography scan showing main bile duct dilation caused by a periampullary duodenal diverticulum, represented by the yellow arrow.

**Figure 2:**
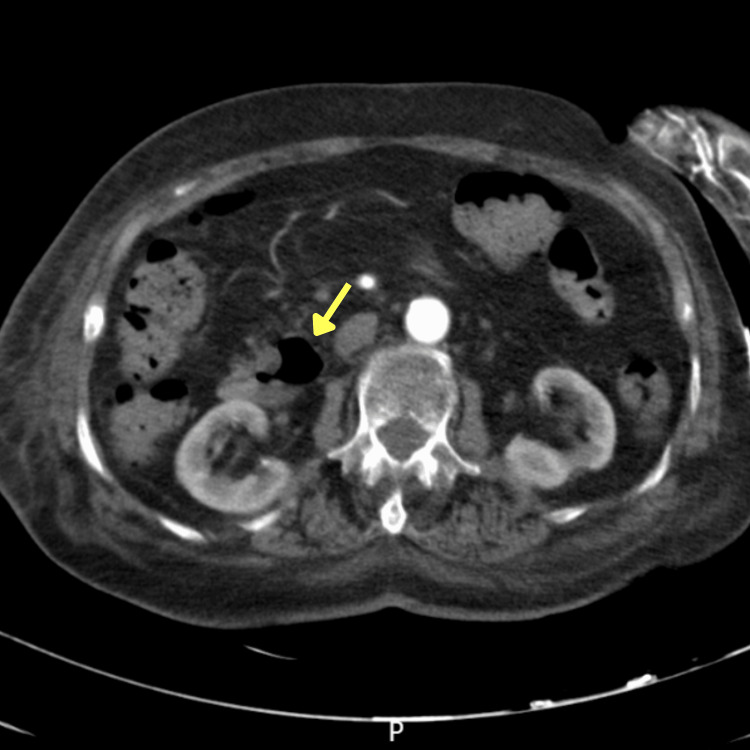
Axial section of an abdominal computed tomography scan showing extrinsic compression of the main bile duct by a periampullary duodenal diverticulum, represented by the yellow arrow.

Due to the patient’s previous functional status, conservative management was decided. The patient died due to complications of a nosocomial pneumonia.

## Discussion

Lemmel syndrome is an often-overlooked cause of jaundice and/or common bile duct dilation, as more prevalent and clinically significant conditions, such as choledocholithiasis or periampullary malignancies, are usually considered first [2].

Even though the majority of PADs do not produce symptoms, complications arise in roughly 5% of patients. These may include bleeding, perforation, choledocholithiasis, cholangitis, jaundice, diverticulitis, pancreatitis, formation of bezoars or enteroliths, and intestinal obstruction, among others. One such complication is Lemmel syndrome, which can lead to hepatobiliary and pancreatic disorders even in the absence of choledocholithiasis or malignancy [4,5].

This case emphasizes the crucial role of imaging in the diagnosis of symptomatic duodenal diverticula. Despite the inherent difficulties associated with diagnosis, recognizing Lemmel syndrome is critical to avoid mismanagement. Ultrasonography may be a helpful exam in the evaluation of the intra- and extrahepatic biliary duct, but a CT scan is the preferred imaging modality for diagnosing PADs that exert pressure on the intrapancreatic segment of the main bile duct [2,6]. Oral contrast allows a better assessment of the diverticulum and whether it compresses the papillary duct, whereas intravenous contrast injection may be necessary to differentiate a PAD from a tumor, abscess, or a biliopancreatic pseudocyst. In addition, intravenous contrast highlights the main biliary duct and the ampulla of Vater, which are compressed in Lemmel syndrome. Magnetic resonance cholangiopancreatography may also be a useful exam to assess obstructive jaundice [7], particularly in patients who do not undergo ECRP or endoscopic examination [2]. Nevertheless, ERCP is considered to be the gold-standard diagnostic test for Lemmel syndrome [1,8].

In the minority of symptomatic cases, patients with Lemmel syndrome due to PAD usually present with right upper quadrant tenderness and jaundice. In most cases, laboratory findings show leukocytosis and increased inflammatory markers, including erythrocyte sedimentation rate and C-reactive protein, along with elevated direct and total bilirubin, liver enzymes, alkaline phosphatase, and gamma-GT. Pancreatic enzyme levels may also be raised when a PAD compresses the ampulla of Vater [9].

Treatment is typically not indicated for asymptomatic individuals, and a conservative approach is preferred for those with mild symptoms. However, because most patients with Lemmel syndrome develop manifestations related to biliary obstruction, such as jaundice or abdominal pain, some form of intervention may be necessary. In such cases, ERCP, besides serving as the gold-standard diagnostic modality, is also the treatment of choice, performed via endoscopic sphincterotomy [8,10]. 

## Conclusions

Although duodenal diverticula are an uncommon cause of an acute abdomen, maintaining a high level of suspicion and performing thorough imaging evaluation can help identify this rare source of abdominal symptoms. In conclusion, Lemmel syndrome should be considered in the differential diagnosis of biliary obstruction when PADs are present.
